# Different iron sources to study the physiology and biochemistry of iron metabolism in marine micro-algae

**DOI:** 10.1007/s10534-013-9688-1

**Published:** 2013-11-27

**Authors:** Hugo Botebol, Robert Sutak, Ivo F. Scheiber, Pierre-Louis Blaiseau, François-Yves Bouget, Jean-Michel Camadro, Emmanuel Lesuisse

**Affiliations:** 1 LOMIC, UMR7621, Centre National de la Recherche Scientifique, Universite Pierre et Marie Curie (Paris 06), 66651 Banyuls/Mer, France; 2 Department of Parasitology, Faculty of Science, Charles University in Prague, Prague, Czech Republic; 3 Institut Jacques Monod, Centre National de la Recherche Scientifique, Universite Paris Diderot (Paris 07), 75013 Paris, France; 4 Institut Jacques Monod, CNRS, Université Paris Diderot, Bât. Buffon, 15 rue Hélène Brion, 75205 Paris Cedex 13, France

**Keywords:** Iron, Marine micro-algae, Ferric citrate, Ferric EDTA, *Ostreococcus*, *Phaeodactylum*, *Emiliania*

## Abstract

We compared ferric EDTA, ferric citrate and ferrous ascorbate as iron sources to study iron metabolism in *Ostreococcus tauri*, *Phaeodactlylum tricornutum* and *Emiliania huxleyi*. Ferric EDTA was a better iron source than ferric citrate for growth and chlorophyll levels. Direct and indirect experiments showed that iron was much more available to the cells when provided as ferric citrate as compared to ferric EDTA. As a consequence, growth media with iron concentration in the range 1–100 nM were rapidly iron-depleted when ferric citrate—but not ferric EDTA was the iron source. When cultured together, *P. tricornutum* cells overgrew the two other species in iron-sufficient conditions, but *E. huxleyi* was able to compete other species in iron-deficient conditions, and when iron was provided as ferric citrate instead of ferric EDTA, which points out the critical influence of the chemical form of iron on the blooms of some phytoplankton species. The use of ferric citrate and ferrous ascorbate allowed us to unravel a kind of regulation of iron uptake that was dependent on the day/night cycles and to evidence independent uptake systems for ferrous and ferric iron, which can be regulated independently and be copper-dependent or independent. The same iron sources also allowed one to identify molecular components involved in iron uptake and storage in marine micro-algae. Characterizing the mechanisms of iron metabolism in the phytoplankton constitutes a big challenge; we show here that the use of iron sources more readily available to the cells than ferric EDTA is critical for this task.

**Electronic supplementary material:**

The online version of this article (doi:10.1007/s10534-013-9688-1) contains supplementary material, which is available to authorized users.

## Introduction

Iron is vital for most living organisms. This element is abundant in the terrestrial environment but often poorly available due to its chemical properties. Iron has a strong tendency to oxidize in aerobiosis to form insoluble precipitates of ferric hydroxides and oxy-hydroxides. As a result, most organisms developed specific and high affinity mechanisms to acquire this element. Iron involved in a Fenton reaction can be toxic and so the specific mechanisms for iron uptake are tightly regulated in most organisms. In terrestrial unicellular eukaryotes, the mechanisms of iron uptake are well documented [reviewed in (Sutak et al. 2008)]. Two main strategies of iron uptake have been described at the molecular level, mostly based on studies of the yeast *Saccharomyces cerevisiae* [reviewed in (Kosman 2003; Philpott and Protchenko 2008; Blaiseau et al. 2010)]. The reductive strategy of iron uptake involves the dissociation of extracellular ferric complexes by reduction and the uptake of ferrous iron through specific or non-specific permeases, or via a high-affinity permease system (Ftr) coupled to a copper-dependent oxidase (Fet). This enables iron to be channeled through the plasma membrane. The nonreductive strategy of iron uptake involves the direct uptake of ferric complexes, without prior dissociation, via specific transporters. The mechanisms of nonreductive iron uptake mostly involve the use of siderophores.

*Chlamydomonas reinhardtii* is a model photosynthetic eukaryotic freshwater organism for the study of iron metabolism which has the same reductive strategy of iron uptake (Merchant et al. 2006; Allen et al. 2007) as yeast. Seawater microorganisms often face very different conditions of iron availability as the transition metal composition of oceans differ greatly from that of terrestrial environments (Butler 1998), and iron levels in surface seawater [for example, in the form of colloidal iron (Wu et al. 2001)] are extremely low (0.02–1 nM) (Turner et al. 2001). It is therefore likely that phytoplankton species use very high affinity uptake systems to capture iron from seawater.

Interest in marine phytoplankton iron uptake mechanisms, and their adaptation to extreme iron scarcity, is increasing due to the importance of phytoplankton in the carbon cycle and in primary oxygen production. The number of species with a sequenced genome is also increasing, facilitating the analysis of the molecular basis of iron uptake [for recent reviews, see (Morrissey and Bowler 2012; Shaked and Lis 2012)]. Different metabolic responses of eukaryotic phytoplankton to iron starvation have been proposed, mainly on the basis of whole genome analyses (Finazzi et al. 2010). Iron uptake models for marine micro-algae have been proposed based on experimental data (Sunda 2001; Shaked et al. 2005; Morel et al. 2008) and on genome analysis (Kustka et al. 2007; Allen et al. 2008; Bowler et al. 2008). Data supports a general model where unchelated iron (Fe′) is taken up directly by cells via a thermodynamically controlled process (Morel et al. 2008). Genome analyses have revealed genes encoding putative proteins which are homologous to the yeast Fre proteins (involved in reductive iron uptake), and models which are similar to the reductive uptake system of yeast have also been proposed (Shaked et al. 2005; Shaked and Lis 2012).

In a recent study of five micro-algae species, we showed that some species were able to reduce iron at the cell surface but that reduction was not a prerequisite for uptake, unlike in yeast (Sutak et al. 2012). Iron binding for uptake is controlled thermodynamically, bound iron then escapes to simple thermodynamic rules (Sutak et al. 2012). Most of the species studied seemed to have both iron uptake systems for ferrous iron and nonreductive uptake systems for ferric iron, independently on their ability to reduce iron (Sutak et al. 2010, 2012). No mechanism allowing the direct uptake of ferric ions without the involvement of siderophores has ever been described in eukaryotic cells. Here, we aim to identify which tools are best to assist the complex task of characterize such systems at the molecular level.

There are several considerations to take into account when considering which iron source should be used to grow marine micro-algae and study iron uptake when iron concentration and availability in the medium must be controlled. Iron should be maintained in a soluble form with a high ferric chelate stability constant (log K_1_): this is required to avoid precipitation of ferric hydroxides and oxy-hydroxides in an aerobic alkaline medium containing high amounts of Ca^2+^ and Mg^2+^ ions (more than 10 mM each) which often compete with iron for its ligands. The ligand/iron ratio can also be increased to push the thermodynamic equilibrium towards the formation of the iron chelate. However, increasing the stability constant of a ferric chelate reduces the pool of unchelated iron (Fe′) in the medium, with the possible consequence of iron becoming unavailable to the cells. This is typically the case when iron is bound to siderophores for which the cells have no specific receptors (Sutak et al. 2012). Ferric EDTA has often been the only source used for studies of iron uptake by marine micro-algae (Anderson and Morel 1982; Shaked et al. 2005; Shaked and Lis 2012). We previously used ferric citrate and ferrous ascorbate as alternative iron sources (Sutak et al. 2010, 2012), but did not systematically compare the benefits and disadvantages of using these sources for different purposes. Here, we compare ferric EDTA with ferric citrate and ferric ascorbate in different experiments to understand when different sources might be preferable. We show that, if ferric EDTA is a good iron source for cell grow, it is a poor source to study the enzymology of iron uptake, the regulation of iron uptake and storage, and to characterize the molecular components involved in these processes. We focused on three algae species exhibiting different characteristics in their iron uptake systems, as previously described (Sutak et al. 2012). The pennate diatom *Phaeodactylum tricornutum* has a ferrireductase system that is induced under iron starvation, the oceanic coccolithophore *Emiliania huxleyi* has no ferrireductase activity at all, and the picoplanktonic prasinophyte *Ostreococcus tauri* has some very low trans-plasma membrane electron transfer activity which is constitutive and does not seem to have a specific role in iron uptake (Sutak et al. 2012). These three species have uptake systems for both ferric and ferrous iron and the following different affinities for iron: *E. huxleyi * >* P. tricornutum* >* O. tauri* (Sutak et al. 2012) (opposite order for their iron requirement for growth). The experiments with these three species illustrate the usefulness of different iron sources readily available to cells and highlights promising methods for further characterization of iron uptake and storage mechanisms in marine micro-algae.

## Materials and methods

### Strains, cell culture and media

Micro-algae were grown at 20 °C under a 12:12 light (3,000 lux) dark regime in a filtered modified f (Mf) medium as described previously (Sutak et al. 2010, 2012). The composition of Mf medium (standard medium used for cell growth) was the following (for 1 l medium): sea salts (Sigma) 40 g (composition: Cl^−^ 19.29 g, Na^+^ 10.78 g, SO_4_^2−^ 2.66 g, Mg^2+^ 1.32 g, K^+^ 420 mg, Ca^2+^ 400 mg, CO_3_^2−^/HCO_3_^−^ 200 mg, Sr^2+^ 8.8 mg, BO_2_^−^ 5.6 mg, Br^−^ 56 mg, I^−^ 0.24 mg, Li^+^ 0.3 mg, F^−^ 1 mg); MOPS 250 mg (pH 7.3); NH_4_NO_3_ 2.66 mg; NaNO_3_ 75 mg; Na_2_SiO_3_.5H_2_O 22.8 mg; NaH_2_PO_4_ 15 mg; 1 ml of vitamin stock (thiamine HCl 20 mg/l, biotin 1 mg/l, B12 1 mg/l); 1 ml of trace metal stock (MnCl_2_.4H_2_O 200 mg/l, ZnSO_4_.7H_2_O 40 mg/l, Na_2_MoO_4_.2H_2_O 20 mg/l, CoCl_2_.6H_2_O 14 mg/l, Na_3_VO_4_.nH_2_O 10 mg/l, NiCl_2_ 10 mg/l, H_2_SeO_3_ 10 mg/l); and 1 ml of antibiotic stock (ampicillin sodium and streptomycin sulfate 100 mg/ml). The Mf medium was buffered with 1 g/l HEPES (pH 7.5). Iron was added in the form of ferric citrate (1:20) or ferric EDTA (1:20). Iron was added as 0.1 μM ferric citrate under standard growing conditions (for routine maintenance of the cultures). We refer to “no iron” medium when no iron was added to the medium; in this condition, we estimated experimentally that the concentration of contaminating iron was less than 1 nM.

Cell growth and chlorophyll fluorescence were determined with a flow cytometer (BD Accury C6). The chemical speciation of iron was estimated using the GEOCHEM-EZ software (http://www.plantmineralnutrition.net/Geochem/Geochem%20Download.htm) (Shaff et al. 2010) and the MINEQL+4.62.2 software (http://www.mineql.com/) (Kraepiel et al. 1999). The algae species used were obtained from the Roscoff culture collection (http://www.sb-roscoff.fr/Phyto/RCC/index.php): *P. tricornutum* RCC69, *O. tauri* RCC745 and *E. huxleyi* RCC1242 (a calcifying strain of *E. huxleyi*).

### Iron uptake assays

Iron uptake by micro-algae was assayed in microtiter plates or in 2 ml micro-centrifuge tubes as previously described (Sutak et al. 2012). Iron uptake assays were performed with concentrated cell suspensions (from 50 to 250 million cells/100 μl) incubated in the Mf medium described above. ^55^Fe (29,600 MBq/mg) was added to the appropriate concentration in the form of ferrous ascorbate, ferric citrate or ferric EDTA. Iron uptake was stopped at certain time points by adding 0.1 mM BPS, 0.15 mM DFOB and 50 mM EDTA (final concentrations) to the cell suspensions and incubating for 2 min. The cells were collected with a cell harvester (microtiter plates) or by centrifugation (micro-centrifuge tubes), and washed three times on the filter or by centrifugation with washing buffer containing strong iron chelators. The composition of the washing buffer was as follows: 480 mM NaCl, 20 mM KCl, 0.1 mM MgCl_2_, 0.1 mM CaCl_2_, 1 mM BPS (Bathophenanthroline sulfonate), 1 mM DFOB (desferrioxamine B), 50 mM EDTA, 1 mM salicyl hydroxamic acid (SHAM) and 10 mM HEPES (pH 7.5). The washed samples were counted for radioactivity in a Wallac 1450 Micro Beta TriLux scintillaton counter. Cell pigments were bleached with sodium hypochlorite before scintillation counting to avoid quenching. Determination of iron storage and binding under various conditions was also carried out using ^55^Fe (29,600 MBq/mg).

### Electrophoresis

Cells were disrupted by sonication and proteins were solubilized with 0.5 % digitonin. Samples were analyzed by blue native PAGE using the Novex Native PAGE Bis–Tris Gel System (3–12 %) according to the manufacturer’s (Invitrogen) protocol. The gels were vacuum-dried and autoradiographed.

## Results

### Iron speciation

The theoretical speciation of iron (0.1 μM), added as ferric citrate (1:20) or ferric EDTA (1: 1.1) in a medium containing 10 mM CaCl_2_ and 10 mM MgSO_4_ at pH 7.5 is as follows [estimations based on the use of the GEOCHEM-EZ software (Shaff et al. 2010)]: 97.45 % of the iron is expected to precipitate when added as ferric citrate (1:20), with only 2.03 % soluble iron-citrate complex and 0.52 % of iron complexed with OH^−^; 88.58 % of the iron would precipitate when added as ferric EDTA (1:1.1, i.e. a 10 % excess of the EDTA ligand), with 10.9 % soluble ferric EDTA complex and 0.52 % of iron complexed with OH^−^. The ligand/iron ratio of ferric EDTA (using 25.1 as the log_K_ for ferric EDTA) has to be increased to twenty for the theoretical concentration of soluble ferric EDTA to increase to 99.93 % total iron (with 0.02 % iron-OH^−^ complex). These theoretical values are, however, difficult to transpose to real experimental conditions. Iron forms different complexes with citrate, the stability constants of which are not precisely determined. For example, estimated stability constants for the monoiron (III) dicitrate complex is in the range of (log) 19.1–38.7 (Silva et al. 2009). Moreover, theoretical speciation values represent concentrations of the different species when the thermodynamic equilibrium is reached, which depends on the kinetic constants of the reactions. We previously determined experimentally that more than 90 % of iron remained soluble in seawater 30 min after addition of 1 μM ferric citrate (1:20) (Sutak et al. 2010). This suggests that the stability constant of ferric citrate used by most speciation software is underestimated and/or that precipitation of iron from ferric citrate to form ferrihydryie and hematite is limited by the kinetic constants of the reactions.

We compared the effects of using ferric citrate and ferric EDTA sources on cell physiology and biochemistry. Both complexes were in a 1:20 stoichiometry and were mostly soluble (as determined experimentally) in our experimental conditions. A third species, ferrous ascorbate (1:100), was used for short-term experiments. Ferrous iron does not form a stable complex with ascorbate as ascorbate continuously reduces ferric iron into the soluble Fe^2+^ species which is re-oxidized by oxygen. Ferrous ascorbate thus forms a redox system that allows iron to remain soluble in the reduced form until the pool of ascorbate is fully oxidized.

### Effect of the iron source on cell yields and chlorophylls

**Fig. 1:**
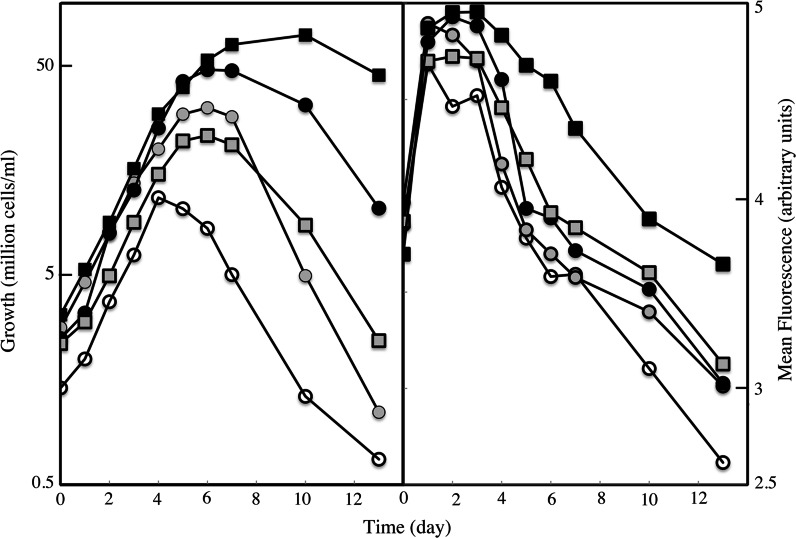
Iron-dependent growth (**a**) and iron-dependent chlorophyll fluorescence (**b**) of *O. tauri*. The cells were precultured for one week in iron-free medium and then inoculated in media containing 0–10 μM ferric citrate (*circles*) or 0–10 μM ferric EDTA (*squares*). Selected *curves* are shown for the following concentrations of iron added to the media: 0 (*empty symbols*), 10 nM (*grey symbols*) and 100 nM (*black symbols*). Values obtained for other iron concentration are presented in Table S1. The cells were grown under a 12:12 light–dark regime, and the number and fluorescence of cells were measured everyday by flow cytometry in the middle of the day. Fluorescence was recorded at ≥670 nm (emission) with excitation at 488 nm (FL3). Data represents mean results from three experiments. Error bars are not shown for the sake of clarity, but SE values were ≤ 9 % for Fig. 1a and ≤ 11 % for Fig. 1b. Full data with SE values are presented in Table S1

We compared ferric EDTA and ferric citrate (1:20) in a wide range of concentrations (1 Nm–10 μM), for their ability to sustain growth of *O. tauri*, *P. tricornutum* and *E. huxleyi*. We also measured the mean fluorescence intensity of chlorophylls (“FL3” channel, excitation 488 nm, emission ≥670 nm), at different time points, as an indication of the amount of cell chlorophylls. Full data are presented in Table S1. Figure 1a shows selected growth curves of *O. tauri* precultured for 1 week in a medium with no iron and then inoculated in media containing various amounts of either ferric EDTA or ferric citrate (full data are presented in Table S1). Cells of this species progressively died a few days after strict iron deprivation (no iron added, Fig. 1a). We had not previously observed this phenotype (Sutak et al. 2012), and this is probably related to both the stringency of the experimental conditions (here, we precultured the cells in iron-free medium) and to the method used to count the cells (this method enables viable cells to be separated from dead cells and cell debris by flow cytometry). In all conditions (ferric citrate or ferric EDTA from 1 nM–10 μM), ferric EDTA was a better iron source than ferric citrate in terms of cell yield. At low iron concentration (1–10 nM), ferric citrate promoted a higher growth rate than ferric EDTA during the first days, and then the cells rapidly died (Fig. 1a). This result suggests that iron was more readily available when provided as ferric citrate than as ferric EDTA, resulting in a boost of growth followed by iron depletion in the medium. At higher iron concentrations (>10 nM) growth rates in exponential growth phase were similar with both mediums but the cells stopped growing earlier when ferric citrate was the iron source (in the range 1–100 nM; Fig. 1a), and the cells then started to die, suggesting that iron became limiting earlier when utilizing ferric citrate. Ferric EDTA also generated higher cell fluorescence intensity scores than ferric citrate for all concentrations of iron tested (Fig. 1b and Table S1): fluorescence intensity increased in a similar way with both sources during the first few days of growth but then decreased more rapidly when using ferric citrate, suggesting again that iron was more rapidly limiting when present as ferric citrate, leading to chlorosis.

We performed the same experiments with the diatom *P. tricornutum* (Fig. S1 and Table S1) and the coccolithophore *E. huxleyi* (Table S1). The growth rates and cell yields of *P. tricornutum* did not differ significantly according to the iron source, but the mean intensity of chlorophyll fluorescence was higher for all the concentrations of iron when using ferric EDTA as the iron source (Fig. S1). No significant difference in growth rates, cell yields or chlorophyll fluorescence was observed with the different iron sources for *E. huxleyi* (Table S1). Overall, we observed that ferric EDTA was a better iron source than ferric citrate for the growth of different marine micro-algae (or equivalent in the case of *E. huxleyi*). The severity of cell preference for ferric EDTA was proportional to the general iron requirements of the species previously determined [*O. tauri* >* P. tricornutum *> *E. huxleyi*; (Sutak et al. 2012)].

### Effect of the iron source on inter-specific competition

**Fig. 2:**
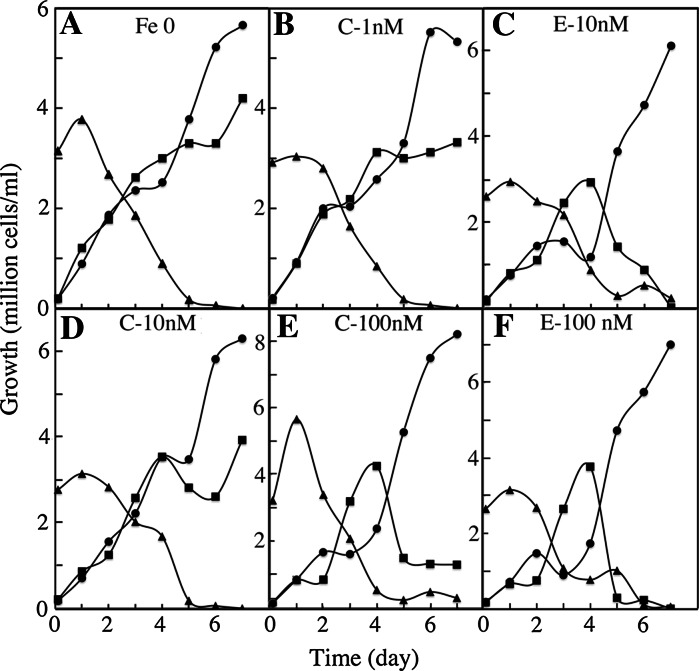
Effect of the concentration and source of iron on the growth of *O.tauri*, *E. huxleyi* and *P. tricornutum* (inter-specific competition). Cells of each species were grown separately for 1 week in iron-free medium and then inoculated together in media containing no iron (Fe 0) or different concentrations (1 nM–10 μM) of ferric citrate (C-XnM) or ferric EDTA (E-XnM). The number of cells of each species in the inoculum was inversely proportional to the estimated value of the cell surface. The cells were grown under a 12:12 light–dark regime, and the number of cells of each species was measured everyday by flow cytometry in the middle of the day. *O. tauri*: triangles; *E. huxleyi*: *squares*; *P. tricornutum*: *circles*. Data are from one representative experiment out of two independent experiments. Other conditions of growth are presented in Fig. S3

Our results suggest that iron availability to the cells is not the same at all points of growth when iron is provided as ferric citrate or as ferric EDTA; iron becomes more rapidly limiting when provided as ferric citrate. This notion is strengthened by inter-specific competition experiments. Cells of the three species were precultured separately for 1 week in iron-deficient medium (no iron added) and then mixed together and grown in media containing ferric citrate or ferric EDTA in the range 1 nM–10 μM. Growth of the three mixed cell populations was measured using flow cytometry on the basis of cell size and fluorescence of each species (Fig. S2). In iron-deficient medium (no iron added), the *O. tauri* population was rapidly outcompeted by the *P. tricornutum* and *E. huxleyi* populations (Fig. 2a). The addition of further iron (as ferric citrate or ferric EDTA) to the medium resulted in complex evolution patterns of the three cell populations (Fig. 2, S3). In iron-rich conditions, the *O. tauri* population decreased continuously to reach zero after a few days, the *E. huxleyi* population showed a transient bloom and then also rapidly decreased to zero, and the *P. tricornutum* population started to increase after a few days until completely outcompeting the two other cell populations (Fig. 2f, S3). This general pattern was observed when the medium contained excess iron (>100 nM) either in the form of ferric citrate or ferric EDTA. However, at lower iron concentrations, there was a clear difference in the evolution of the *E. huxleyi* population with the different iron sources (Fig. 2). When ferric citrate was the iron source, the *E. huxleyi* population continued to increase (1–10 nM iron) or to stabilize (100 nM) alongside the growing *P. tricornutum* population (Fig. 2b, d, e). When ferric EDTA was the iron source, the *E. huxleyi* population stabilized at 1 nM iron (Fig. S3), but rapidly decreased to zero at higher concentrations (Fig. 2b, f). *E. huxleyi* has the lowest iron requirement of the three species (Sutak et al. 2012) which is probably one of the reasons why this species was able to survive together with *P. tricornutum* when no iron was added to the medium (Fig. 2a). This species was able to survive in the presence of the diatom at concentrations of up to 100 nM iron as ferric citrate but not as ferric EDTA, probably because iron depletion of the medium occurred earlier with ferric citrate than with ferric EDTA (supporting our suggestions in the paragraph above).

### Iron uptake from ferric citrate and ferric EDTA

**Fig. 3:**
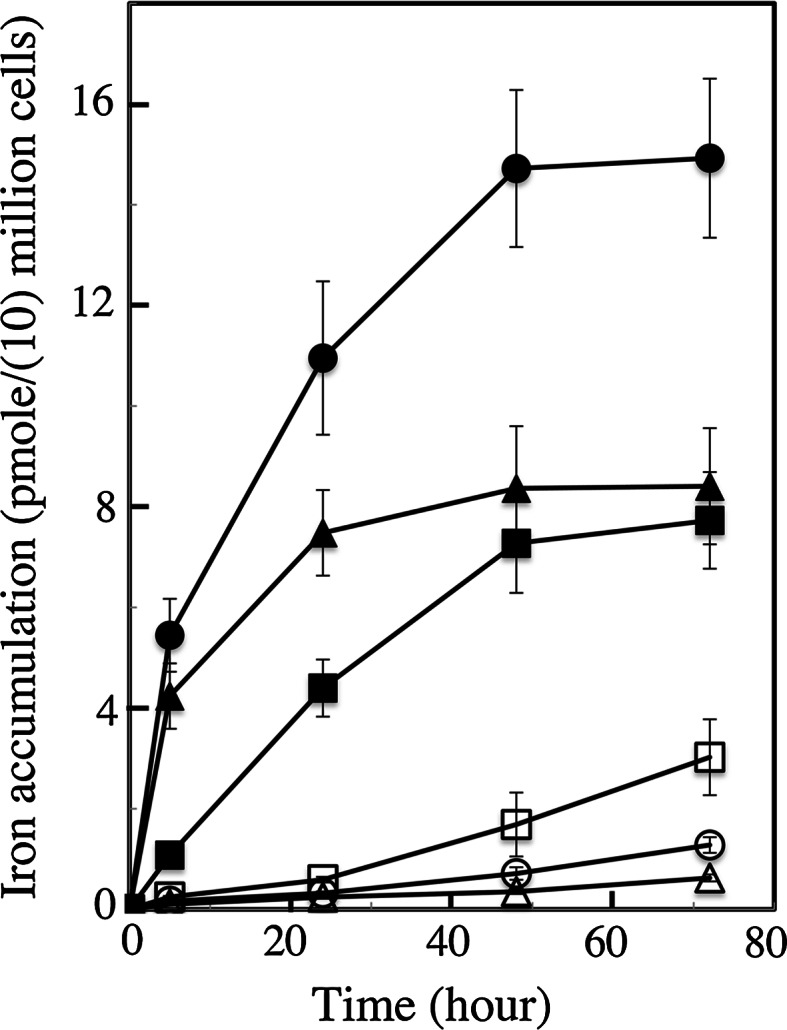
Iron uptake from ferric citrate (*closed symbols*) or ferric EDTA (*open symbols*) during growth of *O. tauri* (*triangles*), *E. huxleyi* (*squares*) and *P. tricornutum* (*circles*). The cells of each species were precultured for 1 week in iron-free medium and then inoculated at 15 million cells/ml (*O. tauri*) or 1 million cells/ml (*E. huxleyi* and *P. tricornutum*) in a medium containing 0.1 μM ^55^Fe-labeled ferric citrate or ferric EDTA (1:20). The cells were grown under a 12:12 light–dark regime. Aliquots of cells were harvested at different points in time during growth, washed three times with a buffer containing strong iron chelators, and the amount of cell-associated iron was determined by liquid scintillation. Results are expressed in p mol/million cells (*E. huxleyi* and *P. tricornutum*) or in p mol/10 million cells (*O. tauri*). Mean±SE from three experiments

In a previous study of short-term kinetics (between 1 and 3 h) under resting conditions (cells suspended in an isotonic buffer), iron was taken up much more rapidly (between 10 and 50 times faster) from ferric citrate than from ferric EDTA (Sutak et al. 2012) in five tested species. Here, we quantified the amount of cell-associated iron during growth in ferric citrate or ferric EDTA mediums. Figure 3 shows the amount of iron associated with each of the three types of cells (and non-removable by washing with strong iron chelators, see methods) during the first 3 d of growth when the cells (1 week iron-free precultures) were inoculated in a medium containing either 0.1 μM ferric citrate or 0.1 μM ferric EDTA. After only two days of growth, *P. tricornutum* and *O. tauri* cells took up more than 90 % of the iron present as ferric citrate in the medium, and only about 2 % of the iron present as ferric EDTA; the values for *E. huxleyi* were about 70 and 10 %, respectively (data not shown). These data strongly support the notion proposed above: when ferric citrate is the iron source for growth, iron is rapidly removed from the medium by the cells in such a way that iron becomes limiting after a few generations, at least when iron is not added in large excess. In contrast, iron is taken from ferric EDTA at a much slower rate, enabling the cells to grow in a medium where the concentration of iron remains nearly constant. This justifies the use of ferric EDTA as an iron source for growth. Several authors have suggested that EDTA buffers an easily calculated pool of unchelated iron (Fe′) in the medium (Shaked et al. 2005; Shi et al. 2010) and allows cells to take up iron from this pool that remains constant throughout growth. The question remaining is as to whether there are any conditions in which other iron sources would be preferable.

### Ferric citrate and ferrous ascorbate as a tool to study the physiology and enzymology of iron uptake

**Fig. 4:**
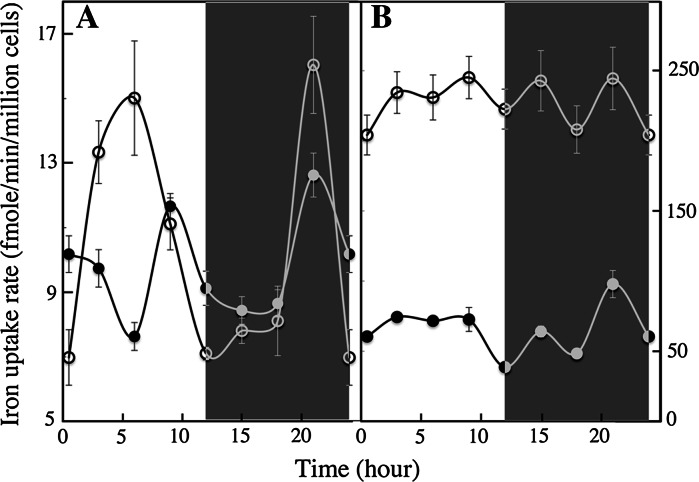
Regulation of iron uptake according to the day/night cycles. *O. tauri* (**a**) and *E. huxleyi* (**b**) cells were grown for 5 d under standard conditions (Mf medium + 0.1 μM ferric citrate) and a 12:12 light–dark regime in two growing chambers programmed in opposition of phase (day started at 10 a.m. in one chamber while night started at 10 a.m. in the second chamber). When the cells were in exponential growth phase, 50 ml of the cultures in both chambers were harvested every 3 h. The cells were washed once with iron-free medium, re-suspended in 1 ml of the same medium and distributed in two micro-centrifuge tubes (2 × 500 μl). ^55^Fe (1 μM) was added as ferric citrate (1:20) (*closed circles*) in one tube and as ferrous ascorbate (1:100) (*open circles*) in the second tube. After 15 min incubation at 20 °C in the light, the cells were washed three times by centrifugation with the washing buffer containing strong iron chelators. Iron associated to the cells was counted by liquid scintillation. *White parts* of the graph shows the iron uptake rates during the day and *dark parts* of the graphs show the iron uptake rates during the night. Mean±SE from three experiments

**Fig. 5:**
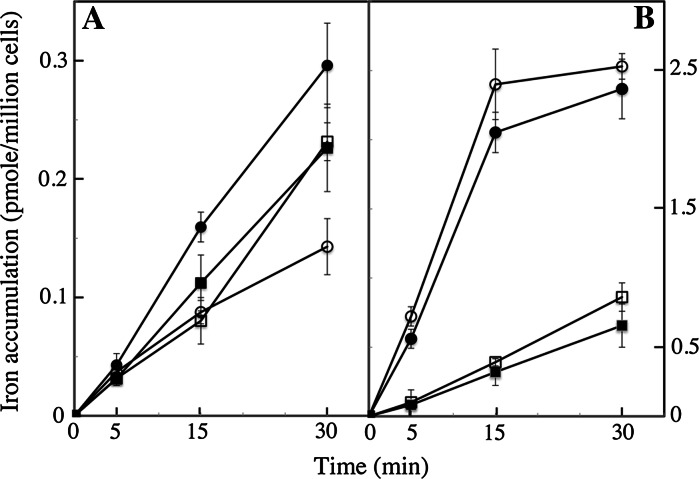
Copper-dependence of iron uptake. *O. tauri* (**a**) and *E. huxleyi* (**b**) cells were grown for 3 d under a 12:12 light–dark regime in Mf medium containing 0.1 μM ferric citrate and either 0.1 μM CuSO_4_ (*closed symbols*) or 0.1 mM of the copper-chelating agent, bathocuproin sulfonate (*open symbols*). Cells were harvested 2 h after dawn, washed once with iron-free and copper-free Mf medium, and tested for iron uptake from 1 μM ferrous ascorbate (*circles*) or 1 μM ferric citrate (*squares*) in microtiter plates (see Sect. 2). Mean±SE from three experiments

To study the mechanisms and regulation of iron uptake by cells, it is essential to measure initial rates of iron uptake. If cells are harvested rapidly at a given moment of their growth, and if iron uptake rates by these cells are measured for a short period of time (10–15 min), the results should reflect the ability of cells to take up iron at this given moment of their growth. This kind of experiment would be difficult to carry out with ferric EDTA, using ^55^Fe with specific activity within the range of commercially available radionuclides (15–100 mCi/mg), as iron uptake from ferric EDTA for short periods of time could be beyond the limit of detection. Moreover, the use of ferric EDTA does not allow one to discriminate between the use of putative ferric and ferrous iron transporters by cells (Sutak et al. 2012). Ferric citrate and ferrous ascorbate may be useful tools to investigate the detailed kinetics parameters of iron uptake and the regulation of iron uptake systems as they can be taken up by cells very rapidly (as shown for *O. tauri* and *E. huxleyi* in Fig. 4). We harvested cells in exponential phase of growth every 3 h over a 24 h day/night cycle (12/12 h), and measured initial (15 min) iron uptake rates by the cells from ferric citrate and ferrous ascorbate. Figure 4 shows that the ability of the cells to take up ferric and ferrous iron varied greatly according to the period of the day or night: peaks of ferrous iron uptake capacity by *O. tauri* occurred in the middle of the day (6 h) and at the end of the night (21 h) (Fig. 4a). Strikingly, the peak of ferrous iron uptake during the day corresponded to the lowest rates of ferric iron uptake. Both ferrous and ferric iron uptake was induced at the end of the night (Fig. 4a). We found no significant change in the capacity of *E. huxleyi* to take up ferrous iron as a function of the day/night cycle, but a significant peak of ferric iron uptake also occurred at the end of the night (Fig. 4b). Results obtained with *P. tricornutum* will be presented in a further study focused on this diatom. These results suggest that some marine micro-algae iron uptake systems are regulated according to the photoperiod, and this is particularly clear for *O. tauri* (Fig. 4a). The results also indicate that ferric and ferrous iron uptake depends on separate systems that are regulated differently. We previously suggested that there might be independent uptake systems for ferrous and ferric iron in the various species studied (Sutak et al. 2012). The present data indicates that it is the case, at least for *O. tauri*. We then carried out a further experiment, which indicated that there are at least two independent iron uptake systems in *O. tauri*. We grew *O. tauri* (and *E. huxleyi*) cells in copper-containing medium (0.1 μM) and copper-depleted medium (medium containing 100 μM of the specific copper chelator, bathocuproin sulfonic acid) for 3 d; the cells were harvested in the middle of the day, washed with copper-free and iron-free medium and used for iron uptake assays with either ferric citrate or ferrous ascorbate. Figure 5a shows that ferrous iron uptake was copper-dependent whereas ferric iron uptake was not. In contrast, neither ferric nor ferrous iron uptake showed copper-dependence in *E. huxleyi* (Fig. 5b).

### Ferric citrate and ferrous ascorbate as a tool to study the biochemistry of iron uptake

**Fig. 6:**
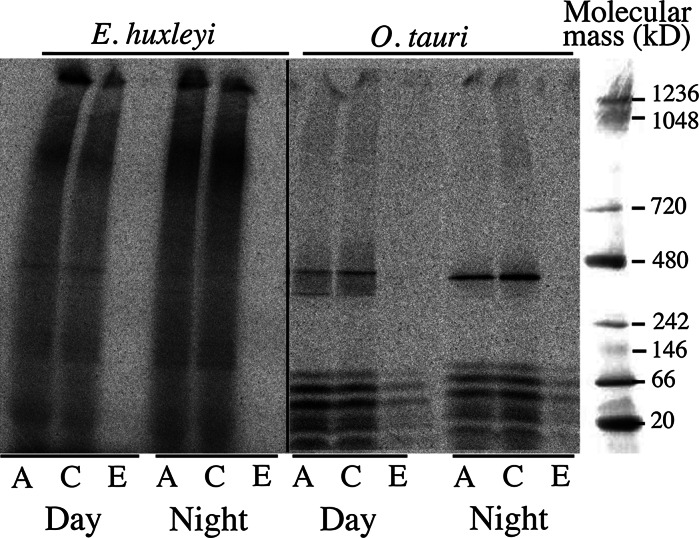
Autoradiography of dried gels after separation of whole cell extracts on blue native PAGE. *O. tauri* and *E. huxleyi* cells were grown for 5 d under standard conditions (Mf medium+0.1 μM ferric citrate) and a 12:12 light–dark regime. Cells in exponential growth phase were harvested in the middle of the day (“Day”) or in the middle of the night (“Night”), washed once by centrifugation with iron-free Mf medium and incubated in the same medium for 2.5 h (*E. huxleyi*) or 1.5 h (*O. tauri*) in the light at 20 °C with either 2 μM ^55^ferrous ascorbate (1:100; “A”), 2 μM ^55^ferric citrate (1:20; “C”) or 2 μM ^55^ferric EDTA (1:20; “E”). Cells were then washed once by centrifugation with iron-free Mf medium (*E. huxleyi*) or (*O. tauri*) with a medium containing strong iron chelators (see Sect. 2), and whole cell extracts were prepared by sonication. After native PAGE (about 25 μg protein per lane), the gels were dried and autoradiographed

We previously showed that iron-binding proteins of marine micro-algae could be identified by mass spectrometry: cells were incubated for various periods of time with ^55^Fe(III)-citrate or ^55^Fe(II)-ascorbate, total protein extracts were prepared and subjected to native gel electrophoresis, and autoradiography of dried gels was used to identify iron-containing bands (Sutak et al. 2012). In the present study, we performed similar experiments and included ^55^Fe(III)-EDTA as an iron source. Cells from different species were harvested during the exponential growth phase either in the middle of the day or in the middle of the night and incubated (in the light) for 1.5–2.5 h in iron-free growth medium supplemented with either 2 μM ^55^Fe(III)-citrate, 2 μM ^55^Fe(II)-ascorbate or 2 μM ^55^Fe(III)-EDTA. The cells were washed and disrupted by sonication before submitting total cell extracts to native gel electrophoresis. Results of autoradiography are shown in Fig. 5 for *E. huxleyi* and *O. tauri*. Our results show that iron from both ferric citrate and ferrous ascorbate was rapidly bound to molecular components of the cells, unlike iron from ferric EDTA (Fig. 6). We previously identified the main band of iron-containing proteins in *O. tauri* as ferritin (Sutak et al. 2012), and this protein was more efficiently loaded with iron when ferrous ascorbate was the iron source than when ferric citrate was the iron source. We obtained the opposite result in the experiment presented in Fig. 6. Iron loading of ferritin was more efficient for cells harvested during the night than for those harvested during the day, and ferric citrate was the preferred iron source in both cases (Fig. 6). When compared with Fig. 4, and with the previously published data (Sutak et al. 2012), the results presented in Fig. 6 suggest that there is no direct relationship between the iron uptake capacity of the cells and the loading of ferritin. In the middle of the day, the ability of *O. tauri* cells to take up ferrous iron was much higher than their ability to take up ferric iron (Fig. 4), but more iron was incorporated into ferritin when ferric citrate was the iron source (Fig. 6).

The pattern of iron-binding components produced by native gel electrophoresis was more complicated for *E. huxleyi* than for *O. tauri* (Fig. 6). A strong background of iron appeared throughout of the gels from which only faint discrete bands could be detected. This background of iron (distributing from the top to the bottom of the gels) was stronger in cells harvested during the night, and was mainly related to specific, non-reversible binding of iron. An important pool of bound iron remained present, even if the cells were washed with strong iron chelators (see Sect. 2) before being disrupted (Fig. S4). In this case, the background of bound iron was stronger when the cells were incubated with ferrous ascorbate than when they were incubated with ferric citrate before washing (Fig. S4). This suggests that iron (especially ferrous iron) is rapidly incorporated into non-protein structures of the cell surface that do not migrate as discrete bands on gels. No background of iron or discrete band appeared when ferric EDTA was the iron source (Fig. 6). Interestingly, a faint band was detected at the same molecular mass as the ferritin band of *O. tauri*. We are currently trying to identify the corresponding protein by mass spectrometry.

This biochemical approach to characterizing the molecular components involved in iron metabolism in marine micro-algae was found to be a powerful assessment tool. Ferric citrate and/or ferrous ascorbate must be used in the place of ferric EDTA for such an approach.

## Discussion

Experimental protocols are often influenced by custom and tradition. The first uptake experiments in baker yeast were performed using ferric citrate and ferrous ascorbate as iron sources (Lesuisse et al. 1987), and these sources have been used in most of the studies that followed. In the field of plant iron metabolism, ferric EDTA has been frequently used (Moog et al. 1995), and is almost exclusively used by oceanographers (Anderson and Morel 1982; Shaked et al. 2005; Shaked and Lis 2012). There is of course a rationale for this choice: seawater contains huge amounts of Ca^2+^ and Mg^2+^ ions, which compete with iron for most of its putative ligands, and so the general problem of iron insolubility in aerobic media is more acute in seawater. It is thus necessary to use iron complexes with relatively high affinity constants to prevent iron precipitation in seawater. It has long been observed that iron uptake by eukaryotic phytoplankton was related to the concentration of unchelated ferric iron species (Fe′) and was independent of the concentration of iron chelated to synthetic ligands (Sunda 2001; Morel et al. 2008). This observation gave rise to a model (called the “Fe′ model”) in which the rate of iron uptake is controlled thermodynamically and is limited by the concentration of unchelated iron (Fe′) in the medium (Morel et al. 2008). In this model, it is critical to control the pool of unchelated iron (Fe′) in the medium. This is easily done using EDTA, because this ligand forms complexes with divalent and trivalent cations which have stability constants that are clearly and precisely defined (Shaked et al. 2005).

Genes encoding putative proteins homologous to proteins involved in yeast iron uptake were identified (Fre, Fet and Ftr proteins) after the genome of several marine micro-algae has been sequenced. This gave rise to new models of iron uptake by marine micro-algae (by diatoms) involving a reduction step and a kinetic control of uptake, as in yeast (Shaked et al. 2005; Allen et al. 2008; Morrissey and Bowler 2012). In this model, it would be paradoxical to continue to use ferric EDTA for iron uptake studies, since the very principle of reductive iron uptake is that the cells dissociate iron from its ligands by reduction at the cell surface: the role of the reduction step is to facilitate ligand exchange (De Luca and Wood 2000). EDTA is one of the very few ligands of iron that forms complexes with both ferric and ferrous iron with very high affinity: the stability constant (log K_1_) of ferric EDTA is 25.7 and the stability constant of ferrous EDTA is 14.3 (in comparison, the respective values are more than 20 for ferric citrate and only three for ferrous citrate (Silva et al. 2009)). In a reductive model of iron uptake, reduction of ferric EDTA would not help the cells to take up iron and so ferric EDTA cannot be used as an iron source by yeasts.

We have already used ferric citrate and ferrous ascorbate to study iron uptake in marine micro-algae (Sutak et al. 2010, 2012). However, we did not systematically compare the benefits and drawbacks of using the different iron sources. In this paper we aimed to establish clear rationale for the use of particular iron sources in order to open new perspectives in the field of iron metabolism in marine micro-algae. This work will be followed by other studies where we will apply the techniques developed here to decipher more specific questions (namely, the mechanisms of iron uptake by diatoms, the role of ferritin in *O. tauri*, the iron-copper connection and the role of iron in inter-specific competition).

Our main conclusion is that ferric EDTA remains the best iron source in terms of growth and cell yield, but is not a good tool to study the enzymology and biochemistry of iron uptake by marine micro-algae. Paradoxically, ferric EDTA is a good iron source for growth because it is a poor iron source for uptake. Marine micro-algae generally face problems of iron scarcity rather than iron excess, thus phytoplankton probably did not develop efficient mechanisms to repress their iron uptake systems when iron is in excess, in the way that most terrestrial organisms did (Sutak et al. 2008). When more iron is available, more is taken up by the cells. Iron from ferric citrate is much more available to cells, but as the cells take up most of the iron present in the medium very rapidly, it soon becomes limiting in the growth medium. We showed this both directly (by measuring growth rates and cell-associated iron in different conditions), and indirectly (by studying the competition between three species in the presence of ferric citrate and ferric EDTA in a wide range of concentrations). These last experiments will need further work to be fully interpreted. The observation that *O. tauri* “lost” when in competition with the two other species is probably related to the fact that it has the lowest affinity iron uptake systems (Sutak et al. 2012). However, we still do not understand why an increase in iron availability causes the species with the highest affinity iron uptake systems (*E. huxleyi*) to die in the presence of the diatom *T. tricornutum*. One notion to be tested is that the diatom dominates in iron-rich medium through the induction of nitrate assimilation (Marchetti et al. 2012). The competition effect was even more pronounced when these only two species were grown together (data not shown). In the presence of *O. tauri*, there was a transient bloom of *E. huxleyi* before dominance by *P. tricornutum*, but this transient increase in the *E. huxleyi* population did not appear when this species was grown with *P. tricornutum* only In that case, *E. huxleyi* was only able to grow with the diatom in iron-deficient medium (<1 nM; data not shown). The behavior of *E. huxleyi* was clearly related to the availability of iron, allowing us to show that ferric citrate was more available as an iron source than ferric EDTA in a micro-environment model. This model will be developed and studied further by our group, using additional species, different iron sources and other sources and amounts of nutrients like nitrogen and phosphate. The iron supply in oceanic high-nitrate, low-chlorophyll environments exerts controls on the dynamics of phytoplankton blooms, which in turn affect the biogeochemical cycles (for example, of carbon, nitrogen, silicon) (Boyd et al. 2007). Large-scale iron fertilization of the ocean was therefore proposed as a possible tool to decrease atmospheric carbon dioxide and help to mitigate climate change (Buesseler and Boyd 2003). Iron fertilization of the ocean can however stimulate growth of toxigenic species (Silver et al. 2010; Trick et al. 2010). As previously reported for *E. huxleyi* (Muggli and Harrison 1996), the bloom of given species depends on several factors which we have shown includes the chemical form of the iron.

We found that the iron uptake systems of marine micro-algae can be regulated according to the day/light cycles. Particular interesting is the observation that *O. tauri* cells to take up ferrous iron best around the middle of the day, when their ability to take up ferric iron is decreasing. As *O. tauri* does not show clear ferrireductase activity (Sutak et al. 2012), this regulation could reflect an adaptation of the cells to facilitate iron uptake after photoreduction, which is expected to be maximal at the middle of the day. Iron naturally reduced by photoreduction might represent a critical pool of iron for some species (Sunda 2001; Sunda and Huntsman 2003). This peak of ferrous iron uptake was neither observed in the coccolithophore *E. huxleyi* nor in the diatom *P. tricornutum* (data not shown; a further study will be devoted to iron uptake by this species), suggesting that this regulation of the ferrous iron uptake system may be specific to some phylogenetic groups. It is worth noting that, whereas *E. huxleyi* has no ferrireductase activity, *P. tricornutum* does have an inducible ferrireductase activity (Park et al. 2011), meaning that photoreduction is not a prerequisite for ferrous iron uptake by this species. *E. huxleyi* will be discussed further below.

The circadian clock regulates genes involved in photo-protection of *O. tauri* cells, in the defense against oxidative stress and the gene encoding ferritin (Monnier et al. 2010). The co-regulation of genes involved in iron uptake could seem plausible as ferrous iron can generate oxidative stress through the Fenton reaction and iron is loaded into ferritin rapidly after its uptake in *O. tauri* [as shown here and in (Sutak et al. 2012)]. Moreover, redox-sensitive proteins and redox-active cofactors are themselves involved in modulating the circadian oscillator and/or relaying the light/dark information to the oscillator in cyanobacteria and terrestrial plants (Dong and Golden 2008; Silver et al. 2010; Carre and Veflingstad 2013). Studying the regulation of iron metabolism as a function of day/night cycles is thus particularly relevant and we are currently working on these specific questions. Such studies could not be undertaken with ferric EDTA as the iron source: forms of iron that are rapidly taken up by the cells and incorporated into proteins and cofactors are required to follow the detailed changes in iron uptake and storage as a function of the moment of the day/night cycle.

Uptake experiments realized with ferric citrate and ferrous ascorbate showed that, at least in *O. tauri*, there are distinct systems for the uptake of ferric and ferrous iron. The ferrous uptake system of *O. tauri*, but not that of *E. huxleyi*, is copper-dependent whereas ferric iron uptake is copper-independent. The iron-copper connection is well documented in yeast, and more generally in terrestrial eukaryotes (Kaplan and O’Halloran 1996). The multi-copper oxidase Fet3 (or functional homologues like ceruloplasmin and hephaestin in human) is required by yeast to re-oxidize iron during its uptake by a mechanism involving the interaction between Fet3 and the permease Ftr1 (Askwith et al. 1994). Little is known about such a putative connection in eukaryotic phytoplankton. Copper-dependence of iron uptake has been shown in diatoms (Maldonado et al. 2006), but the molecular bases of the iron-copper connection in marine micro-algae remain unclear. In yeast, copper-mediated oxidation of iron is part of the iron uptake process itself via a channeling, kinetically controlled mechanism (Kwok et al. 2006). Some phytoplankton species including *O. tauri*, and *P. tricornutum* have genes encoding putative multi-copper oxidases. Homologues of Fet3 could thus play a similar role in marine micro-algae as in yeast (Allen et al. 2008; Morrissey and Bowler 2012). This notion remains questionable, however. The mechanisms of iron uptake by several marine micro-algae is thermodynamically controlled (Morel et al. 2008), at least in a first step of surface iron binding (Sutak et al. 2012). It is therefore unlikely that putative homologues of Fet3 could function in the same way in yeast and in algae since there is no known evidence of a channeling mechanism in marine micro-algae (Sutak et al. 2012). The molecular bases of the iron-copper connection in some phytoplankton species is yet to be understood.

The use of ferric citrate and ferrous ascorbate, unlike ferric EDTA, enables the molecular components that rapidly bind iron in cells of marine micro-algae to be identified by native gel electrophoresis and mass spectrometry. This approach provides future opportunities to study the regulation of ferritin iron loading/unloading and to identify other proteins involved in iron uptake and storage in different species. It has enabled us to study the role of ferritin in *O. tauri* and the molecular mechanisms involved in iron uptake in *P. tricornutum* (these studies will be presented separately). This biochemical approach gave striking results in the case of *E. huxleyi.* Unlike other species which rapidly bind iron to specific proteins (resulting in discrete bands in autoradiography on native gels), *E. huxleyi* seems to rapidly bind iron to molecular components that does not migrate as discrete bands on native gels. This bound iron (especially ferrous iron) is mostly non-exchangeable with strong iron chelators. A strong, non-reversible binding of ferrous iron to the surface of *E. huxleyi* cells has been previously observed (Sutak et al. 2012). Other authors also observed strong iron-binding components in this species (Rodgher et al. 2010) or organic iron-binding components excreted by this species (Boye and van den Berg 2000). The strong iron-binding properties of *E. huxleyi* cells could be part of a strategy used by this organism to take up iron with huge efficiency. No iron binding was observed when ferric EDTA was the iron source. This observation is paradigmatic of the apparent paradox which seems to be specific to marine micro-algae: iron uptake/binding by cells is inversely proportional to the stability constants of the iron complexes and the ligand/iron ratio (and directly proportional to the pool of unchelated iron Fe′), which indicates a thermodynamically controlled step. However, once iron is bound to the cell surface, it escapes simple thermodynamic rules and becomes non-exchangeable, even by strong iron chelators. Understanding the molecular bases of such new iron uptake mechanisms remains a substantial challenge.

## Electronic supplementary material

Below is the link to the electronic supplementary material.

Supplementary material 1 (PDF 4113 kb)
