# Supplementary material for: Different iron sources to study the physiology and biochemistry of iron metabolism in marine micro-algae
Source: Biometals. 2013 Nov 27;27(1):75–88. doi: 10.1007/s10534-013-9688-1 (PMC3905174; doi:10.1007/s10534-013-9688-1)
Supplement: Supplementary file 1 — Supplementary material 1 (PDF 4113 kb) [file 10534_2013_9688_MOESM1_ESM.pdf]

SUPPLEMENTARY MATERIAL

BIOMETALS

DIFFERENT IRON SOURCES TO STUDY THE PHYSIOLOGY AND BIOCHEMISTRY OF IRON METABOLISM IN MARINE MICRO-ALGAE

Hugo Botebol[a§], Robert Sutak[b§], Pierre-Louis Blaiseau[c], François-Yves Bouget[a], Jean-Michel Camadro[c] and Emmanuel Lesuisse[c]

a. Universite Pierre et Marie curie (Paris 06), Centre National de la Recherche Scientifique UMR7621, LOMIC, F-66651 Banyuls/Mer, France.

b Department of Parasitology, Faculty of Science, Charles University in Prague, Czech Republic.

c. Universite Paris Diderot (Paris 07), Centre National de la Recherche Scientifique, Institut Jacques Monod, F-75013 Paris, France.

§ These authors contributed equally to this work

Correspondence should be sent to:

Emmanuel Lesuisse

Institut Jacques Monod, CNRS-Université Paris Diderot, Bât. Buffon, 15 rue Hélène Brion, 75205 Paris cedex 13, France.

Tel. +33 147 278 028

lesuisse.emmanuel@ijm.univ-paris-diderot.fr

SUPP. **Figure S1**

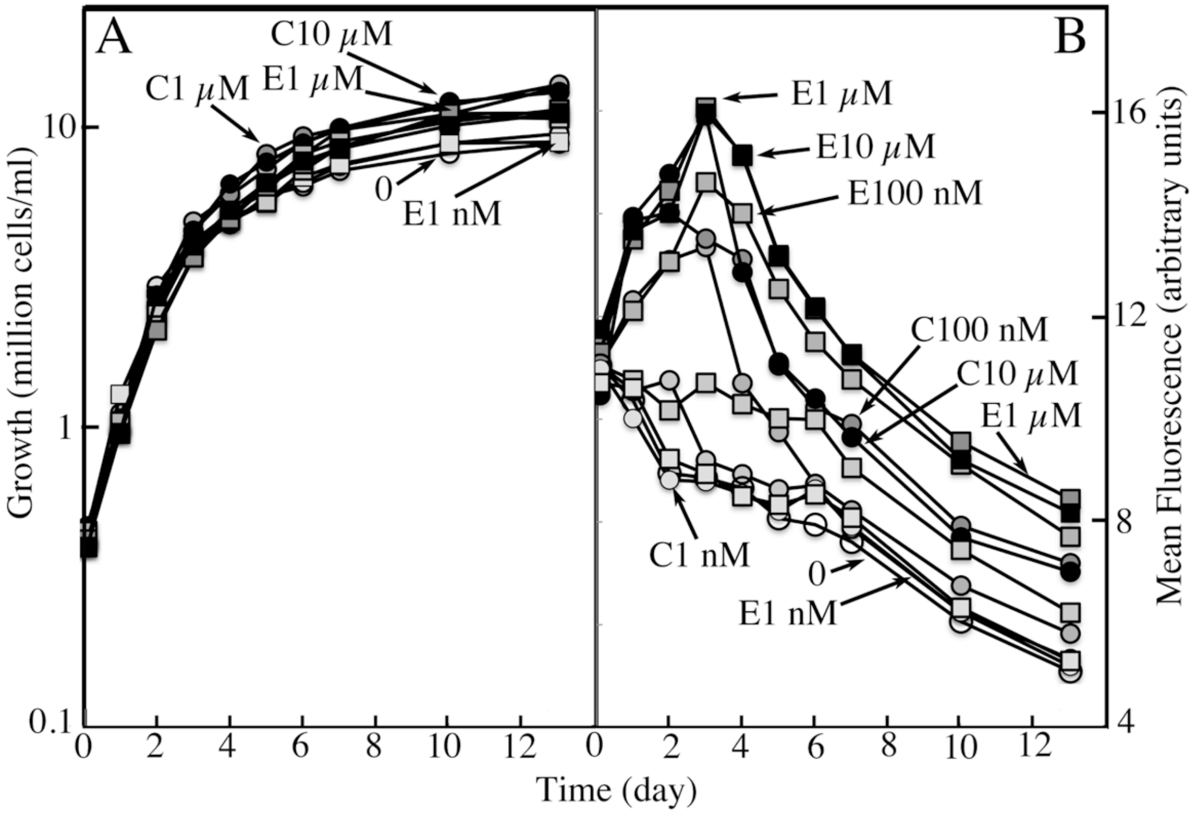

Figure S1. Iron-dependent growth (A) and iron-dependent chlorophyll fluorescence (B) of *P. tricornutum*. The cells were precultured for one week in iron-free medium and then inoculated in media containing 0-10 µM ferric citrate (circles; C-Xn/µM) or 0-10 µM ferric EDTA (squares; E-Xn/µM). The grey scale of symbols (from white to black) represents the increase in iron concentration from 0 to 10 µM. The cells were grown under a 12:12 light-dark regime, and the number and fluorescence of cells was measured everyday by flow cytometry in the middle of the day. Data are from one representative experiment out of two independent experiments (see Table S1).

SUPP. Figure S2

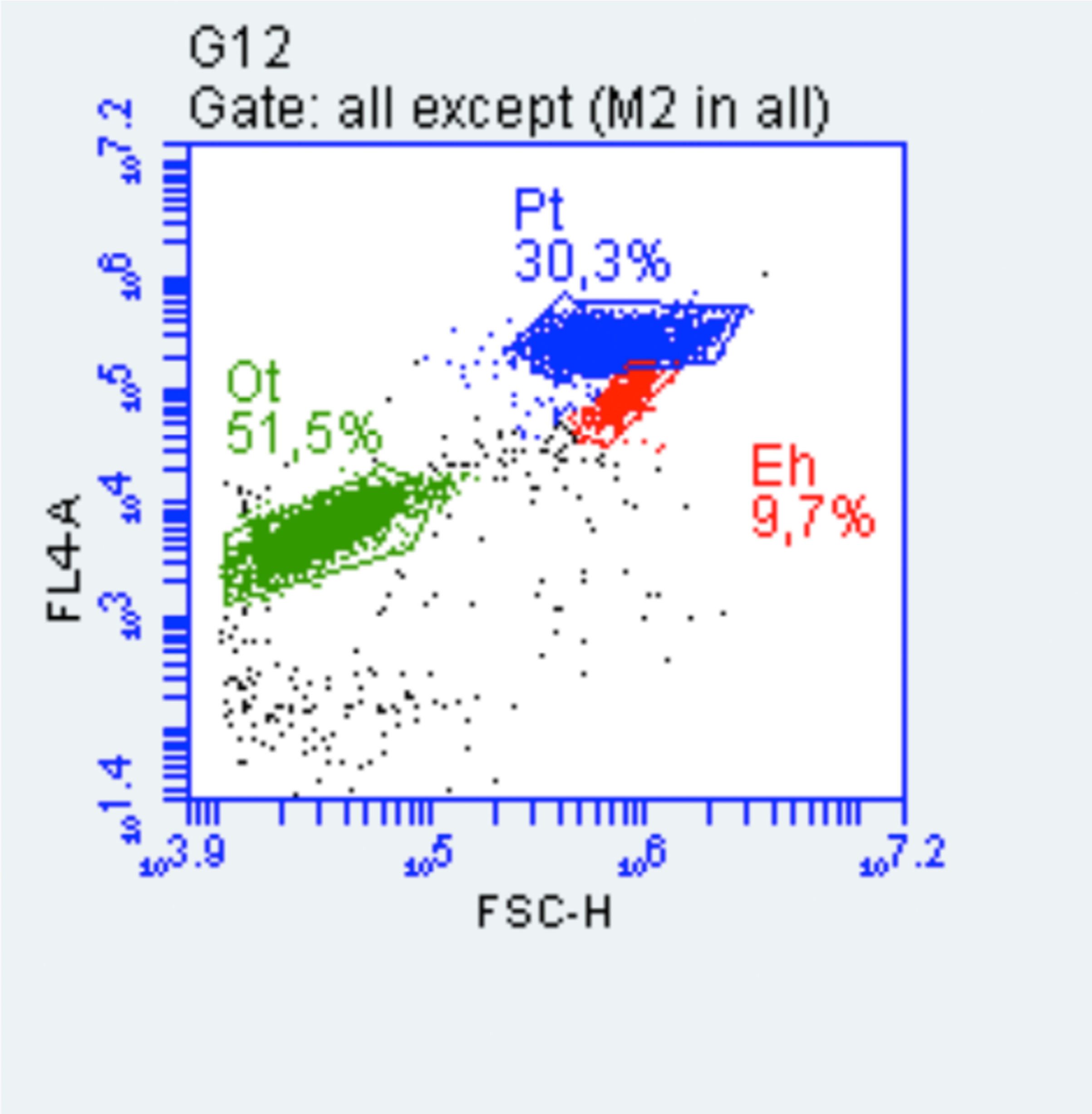

Figure S2. Identification of micro-algae populations by flow cytometry. Populations of *O. tauri* (Ot), *P. tricornutum* (Pt) and *E. huxleyi* (Eh) cells grown together were distinguished on the basis of differential cell fluorescence (FL4 channel: excitation 640 nm, emission 663-687 nm) and differential cell size (FSC-H).

SUPP. Figure S3

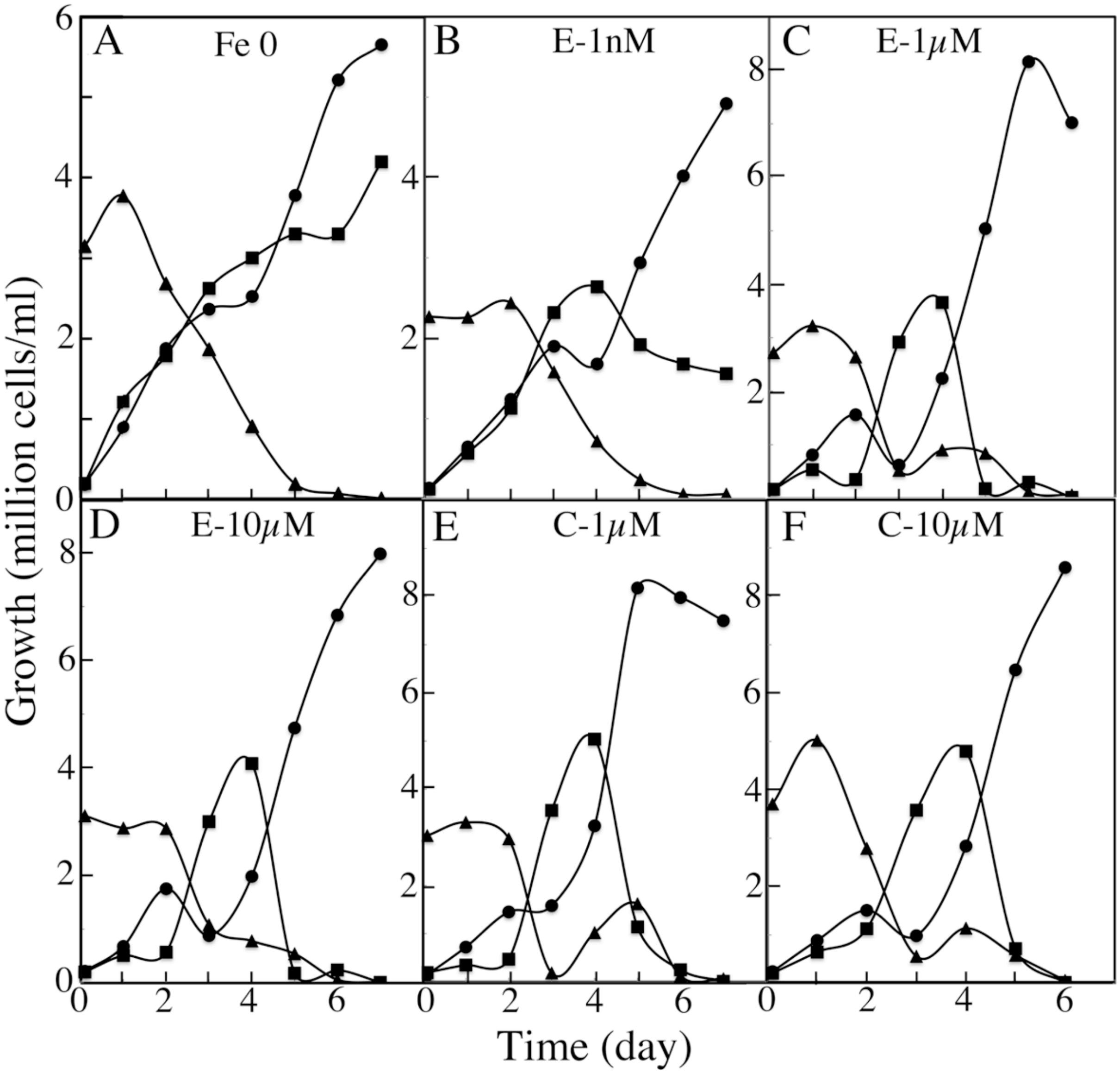

Figure S3. Effect of the concentration and source of iron on the growth of *O.tauri*, *E. huxleyi* and *P. tricornutum* (inter-specific competition). Cells of each species were grown separately for one week in iron-free medium and then inoculated together in media containing no iron (Fe 0) or different concentrations (1nM-10 µM) of ferric citrate (C-XnM) or ferric EDTA (E-XnM). The number of cells of each species in the inoculum was inversely proportional to the estimated value of the cell surface. The cells were grown under a 12:12 light-dark regime and the number of cells of each species was measured everyday by flow cytometry in the middle of the day. *O. tauri*: triangles; *E. huxleyi*: squares; *P. tricornutum*: circles. Data are from one representative experiment out of two independent experiments.

SUPP. Figure S4

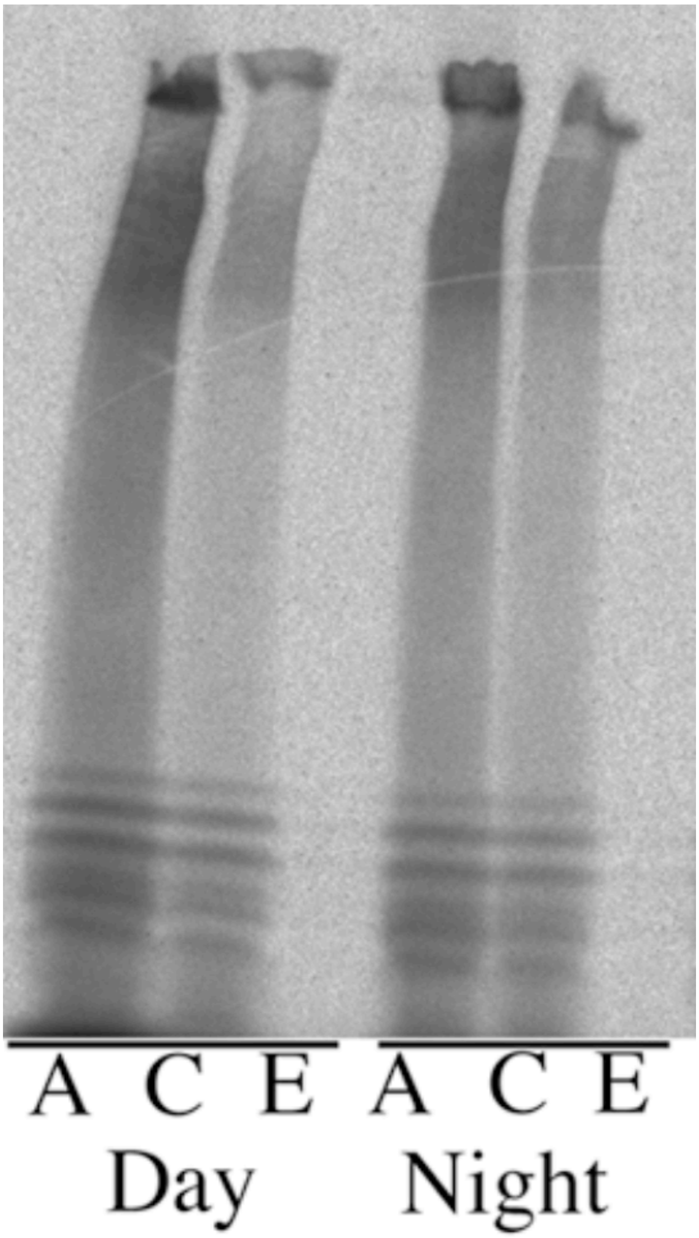

Figure S4. Effect of washing cells with strong iron chelators on bound iron. *E. huxleyi* cells were grown for five days under standard conditions (Mf medium + 0.1 µM ferric citrate) and a 12:12 light-dark regime. Cells in exponential growth phase were harvested in the middle of the day ("Day") or in the middle of the night ("Night"), washed once by centrifugation with iron-free Mf medium, and incubated in the same medium for 1.5 h in the light at 20°C with either 2 µM ^{55}ferrous ascorbate (1:100; "A"), 2 µM ^{55}ferric citrate (1:20; "C") or 2 µM ^{55}ferric EDTA (1:20; "E"). Cells were then washed once with a medium containing strong iron chelators (see methods) and once with iron-free Mf medium. Whole cell extracts were prepared by sonication. After native PAGE (about 25 µg protein per lane), the gels were dried and autoradiographed.

SUPP. Table S1 (A) Mean and SE values (from three experiments) for cell growth and cell fluorescence (FL3) of *O. tauri* cultured with no iron ("0") added and with 1 nM-10 µM ferric citrate ("C-1nM-C-10µM") and ferric EDTA ("E-1nM-E-10µM"). These values correspond to Figure 1. (B) Values (from one representative experiment) for cell growth and cell fluorescence (FL3) of *P. tricornutum* cultured with no iron added and with 1 nM-10 µM ferric citrate or ferric EDTA. These values correspond to Figure S1. (C) Values (from one representative experiment) for cell growth and cell fluorescence (FL3) of *E. huxleyi* cultured with no iron added and with 1 nM-10 µM ferric citrate or ferric EDTA.

A. *Ostreococcus tauri;* growth (Cells/µl; mean)

**Fe**	**0,00**	**C1 nM**	**C10 nM**	**C100 nM**	**C1 µM**	**C10 µM**	**0,00**	**E1 nM**	**E10 nM**	**E100 nM**	**E1 µM**	**E10 µM**
**Time**	Ot1	Ot2	Ot3	Ot4	Ot5	Ot6	Ot7	Ot8	Ot9	Ot10	Ot11	Ot12
**0,01**	1444	1388	2812	2496	2220	2912	1448	1636	2360	3239	1760	2692
**1,00**	3458	3968	4636	3306	4068	5082	1992	3030	2995	5344	3522	4296
**2,00**	3440	4800	7890	8000	8560	6360	3740	4620	4975	8967	7000	10630
**3,00**	7300	7340	13820	12800	15040	14620	6260	6960	8976	16311	12340	15700
**4,00**	12240	15000	20100	25320	26220	31560	11760	13140	15231	29611	32400	36000
**5,00**	11700	12600	29520	42320	47480	39660	10420	15840	21987	39452	67540	72870
**6,00**	9840	9720	31660	48140	68540	83220	8395	12460	23340	53428	117780	138340
**7,00**	9840	7800	28680	47660	98280	145635	5040	7500	21060	63485	137352	147960
**10,00**	2820	2160	4980	32660	58100	158320	1320	2760	8700	70450	93140	118440
**13,00**	900	660	1108	10480	26900	102800	660	480	2437	45345	65268	93340

Ostreococcus tauri; fluorescence (FL3; mean)

**Fe**	**0,00**	**C1 nM**	**C10 nM**	**C100 nM**	**C1 µM**	**C10 µM**	**0,00**	**E1 nM**	**E10 nM**	**E100 nM**	**E1 µM**	**E10 µM**
**Time**												
**0,00**	38484	38418	39654	39707	39248	37969	39411	38654	38675	36923	36694	38070
**1,00**	46835	47797	48946	47978	51860	47992	47904	47312	46987	47999	49749	51852
**2,00**	44639	46955	48345	49288	52360	48005	47705	47016	47234	49498	52241	51264
**3,00**	45200	45023	47012	48817	48469	49393	45437	46032	47127	49543	52219	53493
**4,00**	40442	41765	41649	46036	47127	48395	41608	41591	44569	48227	50060	51946
**5,00**	37802	37882	38234	39345	42644	47175	37791	38133	41890	46456	48059	51146
**6,00**	35808	36628	36987	38851	41691	47143	34798	34505	39123	45851	47547	50820
**7,00**	35926	36384	35765	37154	40257	46262	34874	34264	38345	43403	45041	49445
**10,00**	31033	32222	33976	35148	32934	38782	32015	32269	36013	38791	42567	44996
**13,00**	26154	27093	30123	30268	31307	33974	26768	31438	31268	36392	38538	38972

***Ostreococcus tauri;* growth (Cells/μl; SE from 3 experiments)**

Fe	0	C-1nM	C-10 nM	C-100 nM	C-1μM	C-10μM	0	E-1nM	E-10nM	E-100nM	E-1μM	E-10μM
**Time (day)**												
**0**	21	56	69	78	69	96	26	51	96	123	89	121
**1**	259	359	257	235	389	387	123	236	425	365	159	286
**2**	235	342	652	458	826	524	285	298	589	523	526	895
**3**	489	725	1325	1101	1789	965	356	524	813	1128	869	1259
**4**	1125	1235	1456	1394	2196	2563	1325	968	1596	2547	1986	3002
**5**	1023	1123	2563	3940	3210	2964	1358	1489	2169	2897	3654	4589
**6**	589	895	3022	3987	3529	5248	563	925	1978	3258	6986	6528
**7**	943	742	2159	3869	5698	9563	356	521	1965	3869	10698	9658
**10**	221	205	426	3001	2285	11269	51	231	543	5321	6985	9658
**13**	75	85	124	489	1243	5624	53	25	71	1283	3269	3589

***Ostreococcus tauri*; fluorescence (FL3; SE from 3 experiments)**

Fe	0	C-1nM	C-10 nM	C-100 nM	C-1μM	C-10μM	0	E-1nM	E-10nM	E-100nM	E-1μM	E-10μM
**Time (day)**												
**0**	3569	3987	3658	2968	2569	1356	2506	2690	3698	1962	1952	2569
**1**	4236	5269	5429	4563	4322	3287	1962	4369	3965	5002	3245	3951
**2**	3256	5432	5123	4022	5694	4962	5126	4760	5249	5460	5036	5021
**3**	2695	1235	4198	5063	3188	3941	2964	3964	4022	3612	3901	4972
**4**	4123	3256	3985	3399	4821	5236	3005	4001	3956	2587	4962	5752
**5**	3695	3896	2273	3973	3970	2976	2968	1952	4326	3687	3078	2976
**6**	4125	3573	4322	3978	2321	3332	3002	3201	2290	4026	2263	4832
**7**	3256	4102	4369	4102	1698	2956	3521	3652	3271	4196	3786	3915
**10**	1452	3269	2289	2975	1976	3075	2970	3412	1596	3702	2368	4096
**13**	1963	1429	1398	1965	2356	1256	1496	1369	936	1873	1962	2023

B. Pheodactylum tricornutum; **growth (Cells/μl; from 1 representative experiment)**

**Fe**	**0**	**C-1nM**	**C-10 nM**	**C-100 nM**	**C-1μM**	**C-10μM**	**0**	**E-1nM**	**E-10nM**	**E-100nM**	**E-1μM**	**E-10μM**
**Time (day)**												
**0**	400	412	414	410	424	472	380	456	400	452	424	402
**1**	986	975	1002	1120	985	1080	986	1296	1005	1050	958	966
**2**	2863	2980	2610	2770	2390	2600	2520	2470	2400	2200	2120	2770
**3**	3968	4280	4200	4896	4560	4540	4580	4060	4120	3740	3720	4060
**4**	4800	4862	5220	5986	6060	6540	4620	4920	5040	5100	4980	5340
**5**	5962	5923	6180	7325	8220	7740	5940	5640	5700	6480	6420	6600
**6**	6423	6596	8160	9002	9420	8962	6960	6900	7260	8220	7620	7800
**7**	7236	7523	9900	9965	10040	9952	8460	7625	8700	9060	8580	8580
**10**	8261	8950	11180	11940	12260	12235	11220	8950	10980	10569	11235	10235
**13**	8931	9625	13760	14040	13260	13300	14100	9021	10820	11580	11280	11256

Pheodactylum tricornutum; fluorescence **(FL3; from 1 representative experiment)**

**Fe**	**0**	**C-1nM**	**C-10 nM**	**C-100 nM**	**C-1μM**	**C-10μM**	**0**	**E-1nM**	**E-10nM**	**E-100nM**	**E-1μM**	**E-10μM**
**Time (day)**												
**0**	1104526	1097515	1105827	1106471	1143635	1046812	1070026	1097421	1104734	1107853	1129314	1174758
**1**	1036448	1001272	1054977	1231102	1386549	1393117	1060261	1072807	1075883	1210801	1349941	1366500
**2**	894786	881402	1075624	1309364	1401649	1476961	922642	917628	1017030	1306165	1444279	1400395
**3**	885691	878331	919856	1335107	1351265	1591214	893559	906296	1070483	1460924	1606230	1595180
**4**	865970	854879	892804	1070131	1311245	1285950	850140	887056	1029246	1400371	1512615	1516131
**5**	807059	821361	863462	974748	1104664	1110196	833556	848986	1001554	1253424	1316685	1320259
**6**	794639	863889	870748	874002	1022254	1040097	853697	896842	998987	1150421	1214198	1218656
**7**	761007	791584	786765	822567	990518	964825	808354	854453	905168	1077281	1126508	1123308
**10**	606180	624752	624561	676096	792261	769793	632965	669558	745785	911939	955873	920897
**13**	509174	519068	533128	582848	719757	702932	529514	563913	623466	771094	844288	817343

C. ***Emilliania huxleyi;*** **growth (Cells/μl; from 1 representative experiment)**

Fe	0	C-1nM	C-10 nM	C-100 nM	C-1µM	C-10µM	0	E-1nM	E-10nM	E-100nM	E-1µM	E-10µM
**Time (day)**												
**0**	400	412	414	410	424	472	380	456	400	452	424	402
**1**	986	975	1002	1120	985	1080	986	1296	1005	1050	958	966
**2**	2863	2980	2610	2770	2390	2600	2520	2470	2400	2200	2120	2770
**3**	3968	4280	4200	4896	4560	4540	4580	4060	4120	3740	3720	4060
**4**	4800	4862	5220	5986	6060	6540	4620	4920	5040	5100	4980	5340
**5**	5962	5923	6180	7325	8220	7740	5940	5640	5700	6480	6420	6600
**6**	6423	6596	8160	9002	9420	8962	6960	6900	7260	8220	7620	7800
**7**	7236	7523	9900	9965	10040	9952	8460	7625	8700	9060	8580	8580
**10**	8261	8950	11180	11940	12260	12235	11220	8950	10980	10569	11235	10235
**13**	8931	9625	13760	14040	13260	13300	14100	9021	10820	11580	11280	11256

Emiliania huxleyi; fluorescence **(FL3 X 10^{-3}; from 1 representative experiment)**

Fe	0	C-1nM	C-10 nM	C-100 nM	C-1µM	C-10µM	0	E-1nM	E-10nM	E-100nM	E-1µM	E-10µM
**Time (day)**												
**0**	1105	1098	1106	1106	1144	1047	1070	1097	1105	1108	1129	1175
**1**	1036	1001	1055	1231	1387	1393	1060	1073	1076	1211	1350	1366
**2**	895	881	1076	1309	1402	1477	923	918	1017	1306	1444	1400
**3**	886	878	920	1335	1351	1591	894	906	1070	1461	1606	1595
**4**	866	855	893	1070	1311	1286	850	887	1029	1400	1513	1516
**5**	807	821	863	975	1105	1110	834	849	1002	1253	1317	1320
**6**	795	864	871	874	1022	1040	854	897	999	1150	1214	1219
**7**	761	792	787	823	991	965	808	854	905	1077	1127	1123
**10**	606	625	625	676	792	770	633	670	746	912	956	921
**13**	509	519	533	583	720	703	530	564	623	771	844	817
